# Validation of the adjusted multi-biomarker disease activity score as a prognostic test for radiographic progression in rheumatoid arthritis: a combined analysis of multiple studies

**DOI:** 10.1186/s13075-020-02389-4

**Published:** 2021-01-04

**Authors:** Jeffrey R. Curtis, Michael E. Weinblatt, Nancy A. Shadick, Cecilie H. Brahe, Mikkel Østergaard, Merete Lund Hetland, Saedis Saevarsdottir, Megan Horton, Brent Mabey, Darl D. Flake, Rotem Ben-Shachar, Eric H. Sasso, T. W. Huizinga

**Affiliations:** 1 University of Alabama at Birmingham, 510 20th Street S, Birmingham, AL USA; 2 Divison of Rheumatology, Inflammation and Immunity, Brigham and Women’s Hospital, Boston, MA USA; 3 Copenhagen Center for Arthritis Research and DANBIO, Center for Rheumatology and Spine Diseases, Rigshospitalet, Valdemar Hansens vej 17, Glostrup, Denmark; 4 Department of Clinical Medicine, University of Copenhagen, Blegdamsvej 3B, Copenhagen, Denmark; 5 Division of Rheumatology and Clinical Epidemiology, Department of Medicine, Solna, Karolinska Institutet, SE-171 77 Stockholm, Sweden; 6 Faculty of Medicine, School of Health Sciences, University of Iceland, Reykjavik, Iceland; 7 Myriad Genetics, Inc., 320 Wakara Way, Salt Lake City, UT USA; 8 Crescendo Bioscience, Inc., 180 Kimball Way, South San Francisco, CA USA; 9 Leiden University Medical Center, Albinusdreef 2, 2333 ZA Leiden, Netherlands

**Keywords:** Biomarker, Rheumatoid arthritis, Disease activity, Radiographic progression, Risk prediction

## Abstract

**Background:**

The multi-biomarker disease activity (MBDA) test measures 12 serum protein biomarkers to quantify disease activity in RA patients. A newer version of the MBDA score, adjusted for age, sex, and adiposity, has been validated in two cohorts (OPERA and BRASS) for predicting risk for radiographic progression. We now extend these findings with additional cohorts to further validate the adjusted MBDA score as a predictor of radiographic progression risk and compare its performance with that of other risk factors.

**Methods:**

Four cohorts were analyzed: the BRASS and Leiden registries and the OPERA and SWEFOT studies (total *N* = 953). Treatments included conventional DMARDs and anti-TNFs. Associations of radiographic progression (ΔTSS) per year with the adjusted MBDA score, seropositivity, and clinical measures were evaluated using linear and logistic regression. The adjusted MBDA score was (1) validated in Leiden and SWEFOT, (2) compared with other measures in all four cohorts, and (3) used to generate curves for predicting risk of radiographic progression.

**Results:**

Univariable and bivariable analyses validated the adjusted MBDA score and found it to be the strongest, independent predicator of radiographic progression (ΔTSS > 5) compared with seropositivity (rheumatoid factor and/or anti-CCP), baseline TSS, DAS28-CRP, CRP SJC, or CDAI. Neither DAS28-CRP, CDAI, SJC, nor CRP added significant information to the adjusted MBDA score as a predictor, and the frequency of radiographic progression agreed with the adjusted MBDA score when it was discordant with these measures. The rate of progression (ΔTSS > 5) increased from < 2% in the low (1–29) adjusted MBDA category to 16% in the high (45–100) category. A modeled risk curve indicated that risk increased continuously, exceeding 40% for the highest adjusted MBDA scores.

**Conclusion:**

The adjusted MBDA score was validated as an RA disease activity measure that is prognostic for radiographic progression. The adjusted MBDA score was a stronger predictor of radiographic progression than conventional risk factors, including seropositivity, and its prognostic ability was not significantly improved by the addition of DAS28-CRP, CRP, SJC, or CDAI.

**Supplementary Information:**

The online version contains supplementary material available at 10.1186/s13075-020-02389-4.

## Introduction

The goals of managing patients with rheumatoid arthritis (RA) are to minimize inflammation, joint damage, and disability. To achieve these goals, RA disease activity should be quantitatively assessed on a regular basis, with treatment adjusted as needed to achieve remission or the lowest possible level of disease activity [1, 2]. Ideally, this strategy should be initiated from disease onset because the disability associated with joint damage is irreversible.

Preventing new joint damage remains an important goal for RA patient management. Although biologic agents have reduced the amount of joint damage progression observed in clinical trials and in practice, progression occurs in some patients receiving biologic therapy [3–8]. In addition, biologic therapy is not always initiated promptly in patients with inadequate response to a conventional disease-modifying anti-rheumatic drug (DMARD) [9]. Remission can be difficult to achieve, even with treat-to-target strategies [10], in part from difficulty distinguishing between inflammatory and non-inflammatory symptoms [11]. Moreover, joint damage can occur with synovitis that is inapparent on physical examination [12]. In clinical trials, joint damage is measured with radiographs and specialized scoring systems, such as the van der Heijde modified Sharp score [13]. An increase of five Sharp units per year was found to be the minimal clinically important difference needed for rheumatologists to change therapy [14], and increase > 5 units per year has been used to define rapid radiographic progression in clinical trials [15]. In clinical practice, joint damage progression is not usually assessed quantitatively. It could be useful to have a convenient way to assess the risk for new joint damage without X-rays or advanced imaging.

The probability of developing new joint damage is related to several factors, including a history of joint damage, serologic status, and the level of RA disease activity. Of these factors, only disease activity can be reliably influenced by treatment. Most rheumatologists in the US do not routinely use a composite disease activity measure with joint counts to assess RA disease activity, as the required assessments are time-consuming [16]. Moreover, the components of these measures have shortcomings that limit their ability to predict radiographic progression. Joint counts and global assessments are partially or entirely subjective and can vary between assessors [17], and C-reactive protein (CRP) and erythrocyte sedimentation rate (ESR) are objective but are non-specific and insensitive [18, 19]. In a study of 9135 patients with clinically active RA in rheumatology practices in the US, neither CRP nor ESR was elevated in the majority of patients, including 46% of those with a high Clinical Disease Activity Index (CDAI) [20]. These considerations demonstrate the need for an accurate, objective tool that measures RA disease activity and is prognostic for new joint damage.

The multi-biomarker disease activity (MBDA) blood test measures 12 serum biomarkers, including CRP, to generate a score on a scale of 1–100 that represents the level of disease activity in adult patients with RA [21, 22]. The MBDA score has categories of low (< 30), moderate (30–44), and high (> 44) disease activity [21, 22]. Change in MBDA score correlates with change in clinical disease activity [23]. When assessing change from a moderate or high MBDA score to a later score, the minimally important difference is 8 points [24]. In 2019, the MBDA score was the subject of a systematic review and meta-analysis [25], and the American College of Rheumatology (ACR) disease activity measures working group concluded that the MBDA score was one of 11 RA disease activity measures that fulfilled the minimum standard for regular use [26].

In single-cohort studies, a low MBDA score was associated with little or no radiographic progression over the following the year and high MBDA scores conferred the highest risk for progression [6, 27–29]. The MBDA score was a stronger predictor of risk for radiographic progression than conventional disease activity measures in these studies and in a combined analysis of three cohorts [30].

A recent study of over 300,000 RA patients developed and validated a method for modifying the original MBDA score to adjust it for the effects of the age, sex, and serum leptin concentration, used as a surrogate for adiposity [31]. In an analysis of cohorts from OPERA, a study of treatment-naïve patients with recent onset RA, and BRASS, a US registry of patients with predominantly longstanding RA, the adjusted MBDA score was a stronger predictor of the risk for radiographic progression than the original MBDA score and both were stronger predictors than seropositivity and several conventional measures of disease activity [31]. That analysis is the only one to date which examined the risk for radiographic progression using the adjusted MBDA score.

The goal of the present study was to validate the adjusted MBDA score as a prognostic test for radiographic progression in two cohorts that have not been evaluated previously with the adjusted MBDA score. These two cohorts were then pooled with the previously analyzed OPERA and BRASS cohorts to create a large, diverse combined cohort from two prospective studies and two registries to further evaluate the performance of the adjusted MBDA score, to compare it with other measures, and to generate a risk curve for predicting the probability of radiographic progression in individual patients based on the adjusted MBDA score.

## Methods

### Patient cohorts

Four cohorts were selected for this analysis because of their size (> 100 patients) and availability of requisite clinical, radiographic, and MBDA score patient-level data. These cohorts (Leiden, OPERA, SWEFOT, and BRASS) include patients receiving non-biologic and biologic DMARDs. They have not all been pooled previously to form a single cohort, and the Leiden and SWEFOT cohorts have not been analyzed previously with the adjusted MBDA score. Three of these cohorts (Leiden, OPERA, SWEFOT) have been previously analyzed using the original, unadjusted MBDA score as a prognostic for radiographic progression, both individually [6, 28, 29] and in a combined analysis for which they were selected by a literature review conducted in October 2018 [30]. Cohorts from BRASS and OPERA were previously used to validate the adjusted MBDA score as a prognostic for radiographic progression [31]. For consistency, the same cohorts were included here.

### Clinical and laboratory measures

The present analyses used patient-level data that were collected by the respective parent study (OPERA or SWEFOT) or registry (Leiden or BRASS). Swollen and tender joint counts (SJC, TJC) were based on 28 joints. The Disease Activity Score with 28 joints using CRP (DAS28-CRP) categories employed here for remission/low (≤ 2.67, termed “low” hereafter), moderate (> 2.67 to 4.09), and high (> 4.09) disease activity have been established specifically for DAS28-CRP with thresholds that are lower than those for DAS28-ESR [32]. Because DAS28 results in the OPERA parent trial used ESR, CRP data from OPERA were used to generate DAS28-CRP values for use here. Physician global assessment data were unavailable for the Leiden cohort, so CDAI was analyzed only for the OPERA, SWEFOT, and BRASS cohorts. CDAI categories were low (≤ 10), moderate (> 10 to 22), and high (> 22). CRP data were from the respective clinical laboratories used by OPERA, SWEFOT, and BRASS. CRP data for Leiden came from the high-sensitivity measurement in the MBDA test. The CRP categories employed here were low (≤ 3.0 mg/L), moderate (> 3.0 to 10 mg/L), and high (> 10 mg/L) and are based on the threshold used in the ACR/EULAR definition of Boolean remission (CRP ≤ 10 mg/L) [33] and are consistent with categories used in previous reports [15, 28–30, 34–37]. SJC categories were low (SJC = 0), moderate (SJC = 1 to 9), and high (SJC ≥ 10) and were chosen to emphasize the lower end of the SJC spectrum, where elevated adjusted MBDA scores are of special interest. Serologic status was defined as positive if a patient had tested positive for rheumatoid factor (RF), anti-cyclic citrullinated peptide (CCP) antibodies or both in the clinical laboratory of the respective cohort, and negative if the patient had tested negative for both RF and anti-CCP antibodies.

### Radiographic measures

Radiographs of the hands, wrists, and feet were obtained at baseline and 1 year for Leiden, OPERA, and SWEFOT. Radiographs of the hands and wrists were obtained at baseline and approximately 2 years later for BRASS. For each cohort, radiographs were assessed with the van der Heijde-modified total Sharp score (TSS) by unique, independent assessors who were blinded to clinical data and MBDA scores. As validated previously, TSS data from BRASS were multiplied by 1.6 (448/280) to normalize for the number of joints that would have been scored if radiographs had also included the feet, and they were normalized to a per 365 days rate of change (Δ) in TSS for each patient, based on the time between paired radiographs of a patient [31]. Radiographic progression was defined as ΔTSS > 5 units per year unless specified otherwise.

### Multi-biomarker disease activity score measurement

For each cohort, MBDA scores were determined previously using serum samples that had been collected by the parent study or registry and shipped frozen to the laboratory of the Crescendo Bioscience in South San Francisco, CA, for testing, as has been reported [6, 28, 29, 31]. Serum concentrations of 12 biomarkers (vascular cell adhesion molecule 1, epidermal growth factor, vascular endothelial growth factor, interleukin 6, TNF receptor type I, matrix metalloproteinase (MMP) 1, MMP-3, bone glycoprotein 39 (YKL-40), leptin, resistin, serum amyloid A, and CRP) were measured by electroluminescence-based multiplexed sandwich immunoassays (Meso Scale Discovery, Rockville, MD, USA) and used to determine the original MBDA score with a validated algorithm [22]. All testing used the same types of reagents and instrument as are used for the Vectra® test, which has been commercially available in the USA since 2010. All MBDA scores were determined after the corresponding clinical and radiographic assessments were complete and without knowledge of those results.

For the present study, original MBDA scores were adjusted for age, sex, and leptin concentration using a validated algorithm [31]. The original MBDA score and the adjusted MBDA score both have a scale of 1–100, disease activity categories of low (< 30), moderate (30–44) and high (> 44), and a minimally important difference of 8 points [31]. This adjustment has been in the clinical use in the USA since December 2017. Only adjusted MBDA scores were analyzed in this study.

### Statistical analyses

#### Univariable analysis of the adjusted MBDA score and other potential risk factors as predictors of radiographic progression

Univariable analyses of baseline adjusted MBDA score and clinical and demographic variables were performed to predict radiographic progression over 1 year in the Leiden and SWEFOT combined cohorts and in the four cohorts combined. Linear regression models were developed to predict continuous radiographic progression (∆TSS), where coefficient estimates, 95% confidence intervals (CI), and *p* values were reported. Radiographic progression was analyzed as a binary outcome (∆TSS > 5) using logistic regression models, where odds ratios, 95% CI, and *p* values were reported. In univariable and bivariable (see below) analyses, CRP in mg/L was treated as the natural logarithm (ln) of (CRP + 1). All models contained a random effect on cohort, and *p* values were derived from likelihood ratio tests. As risk discrimination is considered a more appropriate metric for assessing performance of a prognostic test, sensitivity, specificity, positive predictive value, and negative predictive value were not evaluated here [38, 39].

#### Descriptive analyses of association between radiographic progression and adjusted MBDA score

A scatter plot was constructed to display the ΔTSS per year as a function of the adjusted MBDA score at the time of the first radiograph for all patients in the combined cohort. The percentage of patients with ΔTSS > 5 per year was determined for patient groups based on the category of adjusted MBDA score (low, moderate, or high) at the time of the first radiograph for each cohort and the four cohorts combined, without weighting. Rates of progression were also determined for subgroups within the high MBDA category.

#### Direct comparison of the adjusted MBDA score and conventional measures of disease activity as predictors of radiographic progression

For the four cohorts combined, the ability of the adjusted MBDA score to predict radiographic progression (ΔTSS > 5) when combined pairwise with each clinical variable was assessed in bivariable analyses using logistic regression models with a random effect on cohort. Odds ratios, 95% CI, and *p* values derived from likelihood ratio tests were reported. In a subsequent descriptive analysis, patients were cross-classified into nine subgroups, based on low (< 30), moderate (30–44), and high (> 44) adjusted MBDA score categories vs. the low, moderate, and high categories of DAS28-CRP, CRP, SJC, and CDAI, respectively, as defined above. The percentage of patients with radiographic progression (ΔTSS > 5 from baseline to 1 year) was determined for each subgroup.

#### Risk curves to show radiographic progression as a function of adjusted MBDA score as a continuous variable

To estimate the risk of radiographic progression (ΔTSS > 5) as a function of the baseline adjusted MBDA score as a continuous variable, a mixed-effects logistic regression model was fit with radiographic progression as the response variable, the adjusted MBDA score as a predictor with a fixed effect, and the respective cohort of each patient as a predictor with a random effect. The predicted risks for radiographic progression over 1 year and the associated 95% profile likelihood-based confidence intervals were calculated for all adjusted MBDA scores from 1 to 100. This risk relationship was presented graphically as a curve. Additional risk curves were generated with radiographic progression defined as ΔTSS > 2, > 3, and > 4.

## Results

### Cohorts and patients

Four cohorts were analyzed to determine the relationship between the adjusted MBDA score and risk for radiographic progression (Table 1). These cohorts included studies of patients with recent onset, active RA for whom a new treatment was being initiated (OPERA and SWEFOT) and registries of patients, predominantly with established RA, who were receiving ongoing treatment as part of routine care (BRASS and Leiden). Treatments varied between cohorts, according to the respective protocols, and included conventional synthetic DMARDs and biologic DMARDs, which were all TNF inhibitors except for one patient receiving rituximab in the BRASS cohort (Table 1).

**Table 1:** Cohort designs, demographic characteristics and disease measures of patients at baseline

Study/registry^**a**^	BRASS	Leiden	OPERA	SWEFOT	4 cohorts combined
Patients, N	401	163	154	235	953
Type of study	Registry	Registry	RT	RT	Registry and RT
**Inclusion criteria and treatment**					
Previous treatment	DMARDs (non-biologic & biologic)	Non-biologic DMARDs (biologic-naïve)	DMARD-naive	Treatment-naive	DMARDs (non-biologic & biologic)
Symptom duration	Variable	Variable^b^	Early RA (< 6 months)	Early RA (< 1 year)	Early & established RA
Treatment received during period of radiographic evaluation	DMARDs: any non-biologic 89.3%, MTX 50.6%; any biologic 38.7%, anti-TNF 38.4%	Ongoing non-biologic DMARDs (alone or in combination)	MTX monotherapy, MTX + adalimumab; each with IA CS for swollen joints	MTX monotherapy, MTX + SSZ + HCQ, MTX + infliximab	DMARDs: non-biologic and/or biologic (see individual studies)
**Patient characteristics, mean or %**					
Age, years (SD)	56.4 (12.7)	55.2 (13.6)	53.1 (14.2)	55.3 (13.7)	55.4 (13.4)
Female	81.8%	66.9%	67.5%	71.9%	74.5%
Seropositive	75.4%	72.8%	76.6%	77.0%	75.5%
Symptom duration	13.8 years	4.6 years^c^	87.2 days	6.1 months	6.8 years^c^
**Baseline disease activity or radiographic progression, mean (SD)**					
DAS28-CRP	3.9 (1.5)	3.4 (1.3)	5.6 (1.1)	5.4 (1.0)	4.5 (1.6)
Swollen joint count^d^	7.9 (7.0)	2.7 (4.0)	9.5 (6.4)	10.8 (5.3)	8.0 (6.6)
CRP, mg/L	9.4 (21.0)	12.0 (17.2)	31.9 (37.3)	35.4 (38.4)	20.0 (31.1)
Adjusted MBDA score	42.4 (15.6)	45.1 (14.3)	62.0 (16.1)	63.3 (15.6)	51.2 (18.2)
CDAI^e^	22.1 (16.1)	–	32.9 (14.2)	28.0 (10.7)	26.1 (14.9)^e^
TSS	50.0 (81.0)	36.2 (38.5)	4.1 (5.4)	4.7 (8.1)	29.1 (58.9)

*Abbreviations*: *ADA* adalimumab, *CRP* C-reactive protein, *CS* corticosteroids, *DMARD* disease-modifying anti-rheumatic drug, *HCQ* hydroxychloroquine, *IA* intra-articular, *IR* inadequate responder, *MTX* methotrexate, *n/a* not available, *RA* rheumatoid arthritis, *RP* radiographic progression, *RT* randomized trial, *SD* standard deviation, *SDC* smallest detectable change, *TSS* modified Sharp-van der Heijde score, *SSZ* sulfasalazine

^a^Parent study or registry that provided the cohorts analyzed for the relationship between MBDA score and radiographic progression, here and in the cited reports

^b^Upon enrollment in the Leiden Early Arthritis Clinic (EAC), all patients had recent onset RA (< 2 years); time between EAC enrollment and inclusion in the cohort used here was variable

^c^Median time from enrollment in the Leiden EAC to performance of baseline assessments for the present study; this median value was used in the calculation of mean symptom duration for 4 cohorts combined

^d^Swollen Joint Count is based on 28-joint counts

^e^Patient global assessment was unavailable and CDAI not determined for Leiden cohort. For 4 cohorts combined, CDAI is based on OPERA, SWEFOT, and BRASS cohorts only (*N* = 766)

### Validation of the adjusted MBDA score as a prognostic test for radiographic progression and comparison with other measures

To validate the adjusted MBDA score independently of its previous validation in the OPERA and BRASS cohorts combined [30], univariable analyses were conducted for the Leiden and SWEFOT cohorts combined. The adjusted MBDA score had a strong univariable association with radiographic progression as a binary variable (ΔTSS > 5), with an OR of 1.05 (*p* = 5.55 × 10^−8^) (Table 2). Associations were also significant, but less so, for seropositivity (*p* = 1.7 × 10^−4^), CRP (tested as ln[CRP + 1], *p* = 0.0018), baseline joint damage (tested as TSS) (*p* = 0.0023), and DAS28-CRP (*p* = 0.035). Univariable associations were not significant in Leiden and SWEFOT combined for SJC, CDAI, male sex, or age (Table 2). Univariable analyses treating ΔTSS as a continuous variable yielded similar results (Table 2, “ΔTSS continuous”). Similar results were also obtained when analyzing all four cohorts combined (*N* = 953), with smaller *p* values observed for most measures. Male sex, age, and CDAI were non-significant for both the continuous and the categorical analyses of ΔTSS in the four cohorts combined (Table 2).

**Table 2:** Univariable analyses of association of baseline measures with radiographic progression. Univariable linear regression was used to evaluate the association of baseline demographic and disease activity-related variables with degree of radiographic progression, treated as a continuous (ΔTSS) or categorical (ΔTSS > 5) variable. The four cohorts analyzed are OPERA, BRASS, Leiden, and SWEFOT. Results are in descending order of statistical significance

Variable	Leiden and SWEFOT cohorts combined					4 cohorts combined				
	*N*^*a*^	***∆TSS (continuous)***		***∆TSS > 5***		*N*^*a*^	***∆TSS (continuous)***		***∆TSS > 5***	
		*Coefficient*^*b*^ *(95% CI)*	*p value*	*Odds ratio (95% CI)*	*p value*		*Coefficient*^*b*^ *(95% CI)*	*p value*	*Odds ratio (95% CI)*	*p value*
**Adjusted MBDA score**	398	0.11 (0.067, 0.14)	1.9 × 10^−8^	1.05 (1.03, 1.07)	5.5 × 10^−8^	953	0.061 (0.044, 0.076)	2.5 × 10^−13^	1.05 (1.03, 1.06)	2.5 × 10^− 11^
**Seropositivity**^**c**^	299/ 397	2.23 (1.00, 3.46)	4.2 × 10^−4^	4.18 (1.89, 11.1)	1.7 × 10^−4^	719/952	1.47 (0.89, 2.06)	9.9 × 10^−7^	6.20 (2.90, 16.1)	7.0 × 10^−8^
**ln(CRP + 1)**	391	1.01 (0.37, 1.50)	6.6 × 10^− 4^	1.45 (1.15, 1.93)	0.0018	946	0.58 (0.33, 0.83)	4.7 × 10^−6^	1.57 (1.29, 1.91)	6.8 × 10^−6^
**Baseline TSS**	398	0.043 (0.017, 0.062)	2.1 × 10^−4^	1.01 (1.00, 1.02)	0.0023	953	0.0074 (0.0028, 0.012)	0.0018	1.01 (1.00, 1.01)	0.0072
**DAS28-CRP**	391	0.53 (− 0.0075, 0.91)	0.055	1.21 (1.01, 1.56)	0.035	927	0.31 (0.11, 0.50)	0.0026	1.24 (1.05, 1.46)	0.0096
**Swollen Joint Count**	398	0.082 (− 0.017, 0.17)	0.12	1.03 (0.99, 1.07)	0.17	953	0.062 (0.02-0.10)	0.004	1.04 (1.00, 1.07)	0.05
**Male sex**	120/ 398	− 0.61 (− 1.78, 0.57)	0.31	0.89 (0.50, 1.55)	0.69	243/953	− 0.45 (− 1.04, 0.14)	0.14	0.78 (0.47, 1.26)	0.32
**Age**	398	− 0.014 (− 0.053, 0.026)	0.50	0.99 (0.97, 1.01)	0.36	953	− 0.0043 (− 0.024, 0.015)	0.66	1.00 (0.98, 1.01)	0.67
**CDAI**	233	0.016 (− 0.057, 0.09)	0.66	1.00 (0.97, 1.03)	0.81	766	0.014 (− 0.0053, 0.034)	0.15	1.01 (0.99, 1.02)	0.47

*Abbreviations*: *CI* confidence interval, *CRP* C-reactive protein, *DAS28-CRP* Disease Activity Score using 28-joint count and CRP, *MBDA* multi-biomarker disease activity, *ln* natural logarithm, *RF* rheumatoid factor, *TSS* van der Heijde-modified total Sharp score

^a^Patients within the total group that had suitable radiographic data and for whom baseline data were available for the indicated variable. Ratios indicate the number of patients in the indicated category and the total number with data available for assessing that variable

^b^Coefficients for continuous variables (i.e., all variables except seropositivity, male, and smoking status) represent slope of the linear regression line, expressed as units of ΔTSS per one-unit change in the indicated variable

^c^Seropositivity was defined as having tested positive for RF, anti-cyclic citrullinated peptide antibodies or both

### Relationship between adjusted MBDA score and radiographic progression in individual patients

**Fig. 1:**
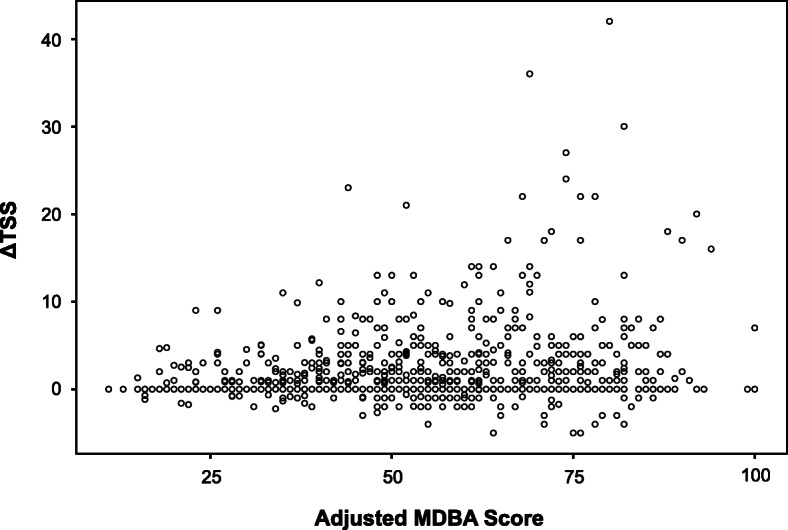
Relationship between baseline-adjusted MBDA score and radiographic progression over the following year. Each open circle represents a pairing of adjusted MBDA score and change in van der Heijde-modified Sharp score (ΔTSS) in the combined cohort (*N* = 953). ΔTSS = 0 for 474 patients. MBDA multi-biomarker disease activity

A scatter plot of data from the four cohorts combined (*N* = 953) illustrated that most progressors had high MBDA scores, and progression (ΔTSS > 5) was nearly absent among patients with low-adjusted MBDA scores (Fig. 1). Radiographic progression tended to be not only more frequent but also more severe among patients with higher adjusted MBDA scores.

### Relationship between category of adjusted MBDA score and rate of radiographic progression

**Fig. 2:**
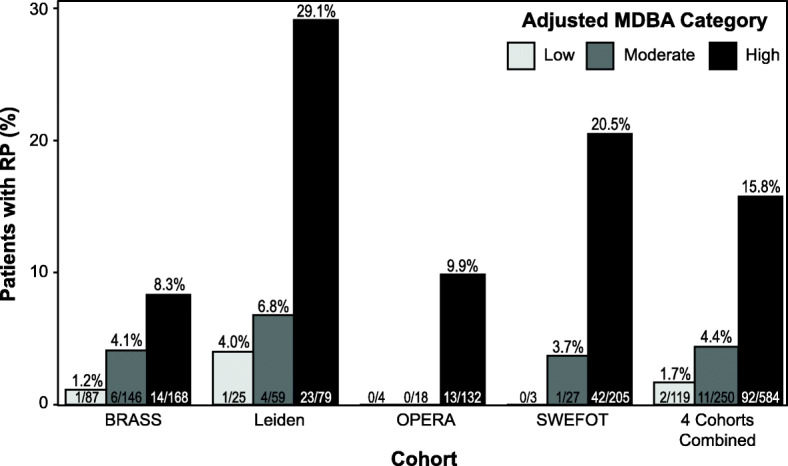
Percentages of patients with radiographic progression (RP) by category of adjusted MBDA score. Results are shown for the Leiden, OPERA, SWEFOT, and BRASS cohorts individually and for the 4 cohorts combined. Radiographic progression was defined as change in van der Heijde-modified Sharp score (ΔTSS) > 5 units per year. Overall, 105 of 953 (11%) patients progressed. MBDA multi-biomarker disease activity

While the four cohorts differed in their disease durations and treatment regimens, each one demonstrated a trend where radiographic progression (ΔTSS > 5 per year) was most frequent in the high adjusted MBDA score category (8.3 to 29.1%) and absent or nearly absent among patients with low-adjusted MBDA scores (0 to 4.0%) (Fig. 2). For the four cohorts combined, radiographic progression was observed in 1.7%, 4.4%, and 15.8% of patients with low (< 30), moderate (30–44), or high (> 44) adjusted MBDA scores, respectively (Fig. 2). For patients with adjusted MBDA scores of 45–60 (*n* = 288) or > 60 (*n* = 296), progression was > 3 Sharp units in 55 (19%) and in 94 (32%) patients, respectively; > 5 Sharp units in 28 (9.7%) and 64 (22%) patients, respectively; and > 10 Sharp units in 7 (2.4%) and 27 (9.1%) patients, respectively.

Risk ratios (95% CI) for ΔTSS > 5 were 2.62 (0.59, 11.6; *p* = 0.24) and 9.37 (2.34, 37.5; *p* = 2.65 × 10^−6^) in the moderate and high-adjusted MBDA score categories, respectively, compared to the low category, and 4.47 (2.54, 7.87; *p* = 5.26 × 10^−10^) for the high category compared to the low and moderate categories combined.

### Bivariable analyses to compare the prognostic value of the adjusted MBDA score vs. other measures

Bivariable analyses found that the adjusted MBDA score added statistically significant information to each other measure for predicting risk for radiographic progression (ΔTSS > 5) (*p* < 1.0 × 10^−6^ for each measure; Supplemental Table 1). By contrast, none of the clinical disease activity measures or CRP added significant predictive information to the adjusted MBDA score (*p* > 0.05 for each), indicating that the adjusted MBDA score fully accounted for their predictive information. Significant predictive information was added to the adjusted MBDA score by seropositivity and, to a lesser degree, baseline TSS, but for each, this additional information was less statistically significant than the information the adjusted MBDA score added to them (Supplemental Table 1). No interaction was found between the adjusted MBDA score and any of the other variables, indicating that the effect of the adjusted MBDA score on radiographic progression was consistent across the levels of each variable.

### Cross-classification of adjusted MBDA score with conventional disease activity measures to evaluate discordances

**Fig. 3:**
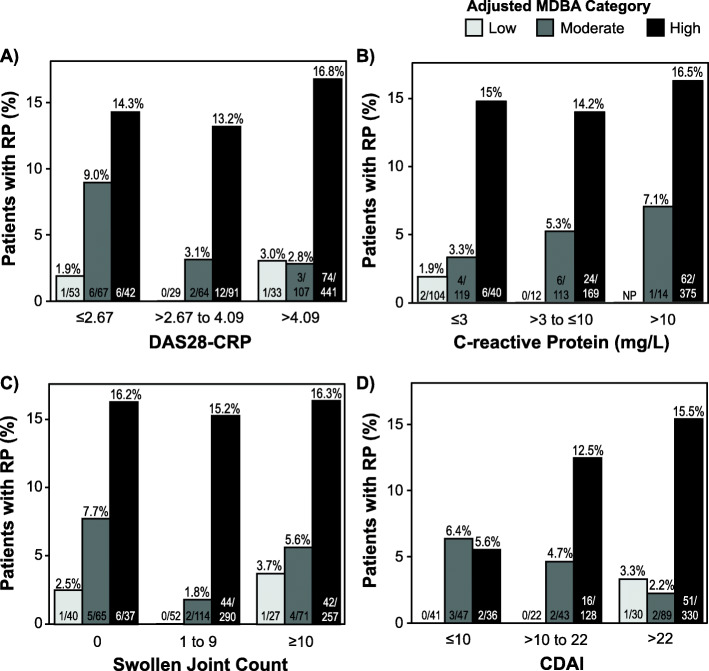
Radiographic progression for patients cross-classified by conventional disease activity measures and adjusted MBDA score. Percentages of patients with progression are shown for patients with low, moderate, and high MBDA scores within the indicated categories of 28-joint disease activity score with CRP (DAS28-CRP) (**a**), C-reactive protein (**b**), 28 swollen joint count (SJC) (**c**), and Clinical Disease Activity Index (CDAI) (**d**). Categories were determined at the time of the first radiograph for each patient. NP no patients. Radiographic progression was defined as change in van der Heijde-modified Sharp score (ΔTSS) > 5 units per year. MBDA multi-biomarker disease activity

To illustrate the finding of the bivariable analyses—that the adjusted MBDA score predicted progression risk independently of the level of clinical disease activity—patients were grouped into low, moderate, and high categories of DAS28-CRP and, within each category, sub-grouped by category of adjusted MBDA score. The frequency of radiographic progression was very low (0–3.0%) when the adjusted MBDA score was low and was highest (13.2–16.8%) when the adjusted MBDA score was high, regardless of whether the DAS28-CRP was low, moderate, or high (Fig. 3a). This type of trend was not observed across DAS28-CRP categories within adjusted MBDA score categories. Analyses of the four cohorts individually, with cross-classification by DAS28-CRP and MBDA score, were generally consistent with the combined cohort analysis, but they should be interpreted with caution, due to the smaller numbers of patients and progressors (Supplemental Figure 1). Similar results were obtained in the combined cohort when cross-classification used categories of CRP, SJC and CDAI (Fig. 3b, c, and d, respectively), except within the low CDAI group, possibly due to the limited number of progressors. Thus, when the adjusted MBDA score was discordant with DAS28-CRP, CRP, SJC, or CDAI, either because the adjusted MBDA score was high and the comparison measure was low or vice versa, the frequency of radiographic progression corresponded more consistently with the category of adjusted MBDA score than the category of DAS28-CRP, CRP, SJC, or CDAI (Fig. 3).

### Risk curves for the MBDA score as a continuous variable for predicting risk of radiographic progression

**Fig. 4:**
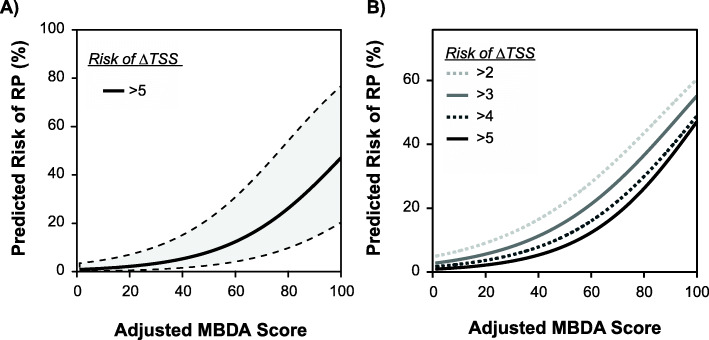
Curves for predicting risk of radiographic prediction as a function of the adjusted MBDA score as a continuous variable. Progression was defined as change (Δ) in van der Heijde-modified Sharp score (TSS) > 5 units per year and shown with 95% confidence interval (**a**), and as ΔTSS > 2, 3, 4, and 5 units per year (**b**). Curves were determined by logistic regression in the combined cohort (*N* = 953). MBDA multi-biomarker disease activity

Because the adjusted MBDA score was the strongest single, independent predictor of radiographic progression (Table 2, Supplementary Table 1), we used logistic regression in the combined cohort to generate a risk curve for radiographic progression (ΔTSS > 5). The probability of progression was lowest when the adjusted MBDA score was low, and it increased continuously as the adjusted MBDA score increased, with an upswing as the score entered the high range (> 44) (Fig. 4a). Among the highest adjusted MBDA scores, the risk of ΔTSS > 5 exceeded 40%. As joint damage is cumulative, we also assessed lower ΔTSS thresholds. Risk of ΔTSS > 4 was slightly greater than for ΔTSS > 5, and the risk of ΔTSS > 3 or > 2 exceeded 50% for the highest MBDA scores (Fig. 4b).

## Discussion

This study has validated the adjusted MBDA score as a prognostic test for radiographic progression in RA using two cohorts that have not been evaluated previously with the adjusted MBDA score. These cohorts were then combined with two others for the largest analysis to date of the adjusted MBDA score as a prognostic test for radiographic progression. These cohorts came from two registries and two clinical trials and were diverse in their levels of clinical disease activity and treatments, which included non-biologic and biologic DMARDs (Table 1). The study produced, for the first time, a risk curve showing the probability of rapid radiographic progression (ΔTSS > 5) across the spectrum of adjusted MBDA scores. A low-adjusted MBDA score (< 30) was associated with very low risk for radiographic progression, with risk increasing continuously as the MBDA score increased and exceeding 40% for patients with the highest MBDA scores.

The adjusted MBDA score was shown to be a more statistically significant predictor of radiographic progression than serologic status, baseline TSS and all of the RA disease activity measures we assessed. While statistically significant univariable differences do not themselves establish clinical meaning, neither DAS28-CRP, CRP, SJC, nor CDAI added significant prognostic ability when directly compared to the adjusted MBDA score in bivariable analyses, whereas the adjusted MBDA score added significant information in each comparison. This finding has two important implications. First, by demonstrating that the adjusted MBDA score may be used independently of clinical assessment to stratify risk for joint damage progression in individual RA patients, it implies that when the adjusted MBDA score is low, the current RA therapy should be adequate for preventing joint damage, whereas, when it is high, a more effective treatment regimen may be needed to reduce the risk for progression.

The second implication of the association between the adjusted MBDA score and radiographic progression is that it supports the adjusted MBDA score as an accurate measure of the type of inflammatory disease activity that damages joints, even when it is discordant with clinical assessments. This property was established with bivariable analyses and was illustrated with cross-classification analyses for both types of discordance: (1) high-adjusted MBDA score with low clinical disease activity, as was observed in 25% of patients with low DAS28-CRP and might occur in patients who have subclinical synovitis or are difficult to examine due to obesity or osteoarthritis and (2) low-adjusted MBDA score with high clinical disease activity, as was observed in 6% of patients with high DAS28-CRP and might occur in patients with fibromyalgia or other forms of non-inflammatory pain (Fig. 3a). Similar results were obtained when the adjusted MBDA score was compared with SJC or CDAI (Fig. 3c, d).

Many studies have found CRP to be a predictor of risk for radiographic progression [40], but its clinical utility is limited because it is often not elevated in patients with clinically active RA [20]. We found that the adjusted MBDA score was a stronger predictor of radiographic progression than CRP and that, in bivariable analyses, CRP added no independent information to the MBDA score. The adjusted MBDA score predicted risk for radiographic progression not only when CRP was low (< 3.0 mg/L), but also when it was intermediate (3–10 mg/L) or higher (> 10 mg/L) (Fig. 3b). This finding is novel and suggests that the superiority of the adjusted MBDA score over CRP as a prognostic for radiographic progression derives not only from its ability of to identify patients with inflammation in the lowest CRP group [34], but also to identify patients who, despite having an elevated CRP, have low risk for radiographic progression.

The CRP results suggest that the MBDA score detected patient heterogeneity that was independent of CRP, which may reflect the role of the 11 non-CRP biomarkers in the MBDA score algorithm [6, 22]. A prior analysis of the SWEFOT study showed that patients with baseline CRP ≤ 10 mg/L and a high baseline MBDA score (> 44) had comparable clinical and radiographic outcomes to those with CRP > 10 mg/L at baseline [41]. The authors concluded that recruitment for trials requiring an elevated CRP may be enhanced by an enrollment criterion of CRP > 10 mg/L and/or MBDA score > 44. The present findings provide additional evidence that the adjusted MBDA score may be complementary to CRP as an enrollment criterion.

A strength of this study is the diversity of the large combined cohort. By including patients with early and established RA and patients treated with non-biologic and biologic DMARDs, the results may be broadly applicable. Access to patient-level data allowed us to calculate adjusted MBDA scores for all four cohorts. The AMPLE study was not included here because patient-level data were not available [42, 43]. However, a reanalysis of published data from AMPLE showed that the associations between the original MBDA score and radiographic progression in the abatacept and adalimumab arms were similar to associations observed in other studies and to what was seen here with the adjusted MBDA score [30, 44].

The purpose of the present study was to validate the MBDA score as a predictor of risk for radiographic progression and compare it with other measures. The ultimate goal of preventing radiographic progression is to maintain physical function, which was not evaluated in this study. The relationship between radiographic progression and physical function, which can be assessed with the Health Assessment Questionnaire (HAQ), typically requires observation over many years to become apparent. In 1-year observations, as analyzed here, HAQ score is more likely to be affected by changes in disease activity than by new joint damage, especially in studies of active, recent-onset RA, like OPERA and SWEFOT. Disease activity contributes to functional decline via a direct effect, from signs and symptoms, and an indirect effect, which is only partly mediated by radiographic joint damage [45]. The MBDA score has been shown to correlate with the HAQ score [23]. The relationship between MBDA score and subsequent change in HAQ score is potentially complex and could be an interesting subject for future investigation.

Other limitations of the present study are that radiographs were assessed by different readers in each cohort, patient global assessments were unavailable for the Leiden cohort, and, except for one patient, TNF inhibitors were the only biologic drugs included in the four cohorts. Data on smoking were not evaluated here [46], but a prior analysis of the SWEFOT cohort found that the original MBDA score was a strong independent predictor of progression (ΔTSS > 5) after adjusting for current smoking status [28].

## Conclusion

In conclusion, we have validated the adjusted MBDA score and performed the largest combined analysis to date of it as a prognostic test for radiographic progression in RA. The adjusted MBDA score was a stronger predictor of radiographic progression than DAS28-CRP, CRP, SJC, and CDAI, and its prognostic ability was not improved by any of these other measures, including when it was discordant with them. A risk curve was generated to show that the risk of rapid radiographic progression approached zero when the adjusted MBDA score was low, and it increased continuously with the adjusted MBDA score, such that risk exceeded 40% and included the most severe cases of progression when the adjusted MBDA score was very high. The results of this study validate the adjusted MBDA score as an objective, independent measure of disease activity that, without requiring information from clinical assessment, can stratify RA patients according to their risk for developing new joint damage.

## Supplementary Information

**Additional file 1 : Supplemental Table 1.** Bivariable logistic regression predicting radiographic progression (∆TSS>5) using adjusted MBDA score with other predictors (DAS28-CRP, CDAI, SJC, seropositivity, baseline van der Heijde modified Sharp score [TSS], log[CRP+1]). Odds ratio (OR) was calculated with 95% confidence interval (CI) and p-value from likelihood ratio test. All models include a random effect on cohort. **Supplemental Figure 1**. Radiographic progression for patients in individual cohorts cross-classified by DAS28-CRP and adjusted MBDA score.

## Data Availability

The datasets used and/or analyzed during the current study are available from the corresponding author on reasonable request.

## Abbreviations

MBDA: Multi-biomarker disease activity
RA: Rheumatoid arthritis
TSS: Total Sharp score
DAS28-CRP: Disease Activity Score with 28 joints using CRP
CRP: C-reactive protein
SJC: Swollen joint count
CDAI: Clinical Disease Activity Index
DMARD: Disease-modifying anti-rheumatic drug
ESR: Erythrocyte sedimentation rate
RF: Rheumatoid factor
CCP: Cyclic citrullinated peptide
MMP: Matrix metalloproteinase
CI: Confidence interval
EAC: Early Arthritis Clinic
ABA: Abatacept
ADA: Adalimumab
CS: Corticosteroids
HCQ: Hydroxychloroquine
IA: Intra-articular
IR: Inadequate responder
MTX: Methotrexate
RT: Randomized trial
SSZ: Sulfasalazine
