# Supplementary material for: Validation of the adjusted multi-biomarker disease activity score as a prognostic test for radiographic progression in rheumatoid arthritis: a combined analysis of multiple studies
Source: Arthritis Res Ther. 2021 Jan 4;23:1. doi: 10.1186/s13075-020-02389-4 (PMC7784276; doi:10.1186/s13075-020-02389-4)

**SUPPLEMENTAL MATERIAL**

**Supplemental Table 1.** Bivariable logistic regression predicting radiographic progression (∆TSS>5) using adjusted MBDA score with other predictors (DAS28-CRP, CDAI, SJC, seropositivity, baseline van der Heijde modified Sharp score [TSS], ln[CRP+1]). Odds ratio (OR) was calculated with 95% confidence interval (CI) and p-value from likelihood ratio test. All models include a random effect on cohort.

|  | **Bivariable Analysis Predicting ∆TSS>5** | | | | | | |  |  |
| --- | --- | --- | --- | --- | --- | --- | --- | --- | --- |
|  |  | | **Adjusted MBDA Score** | | | **Clinical Variable** | | |  |
| **Clinical Variable** | **N** | **OR (95% CI)** | | **p-value** | **OR (95% CI)** | | **p-value** | | |
| **DAS28-CRP** | 927 | 1.05 (1.03, 1.07) | | 9.51x10^-10^ | 0.94 (0.78, 1.14) | | 0.53 | | |
| **ln(CRP+1)** | 946 | 1.06 (1.03, 1.08) | | 9.35x10^-7^ | 0.86 (0.63, 1.16) | | 0.32 | | |
| **SJC** | 953 | 1.05 (1.03, 1.06) | | 1.76x10^-10^ | 1.00 (0.96, 1.04) | | 0.90 | | |
| **CDAI** | 766 | 1.05 (1.03, 1.07) | | 3.56x10^-8^ | 0.99 (0.97, 1.01) | | 0.25 | | |
| **Seropositivity** | 952 | 1.05 (1.03, 1.06) | | 9.48x10^-11^ | 6.00 (2.77, 15.7) | | 2.82x10^-7^ | | |
| **Baseline TSS** | 953 | 1.05 (1.03, 1.06) | | 1.09x10^-10^ | 1.00 (1.00, 1.01) | | 0.037 | | |

Odds ratios represent the factor by which the risk of radiographic progression (∆TSS>5) changes with a one-unit increase in the respective variable. An OR of 1.05 means that for an increase in adjusted MBDA score of 8 units (i.e., the minimally important difference^25^), the OR equals 1.48 (i.e., 1.05^8^); and for an increase of 16 units, the OR equals 2.18.

**Supplemental Figure 1.** **Radiographic progression for patients in individual cohorts cross-classified by DAS28-CRP and adjusted MBDA score.** Percentages of patients with radiographic progression are shown for patients with low, moderate and high adjusted MBDA scores within the low, moderate and high categories of 28-joint disease activity score with CRP (DAS28-CRP). Results are shown separately for patients from the BRASS and Leiden registries and the OPERA and SWEFOT trials of patients with recent onset active rheumatoid arthritis. Categories were determined at the time of the first radiograph for each patient. Radiographic progression was defined as change in van der Heijde modified Sharp score (ΔTSS) >5 units per year. NP, no patients.


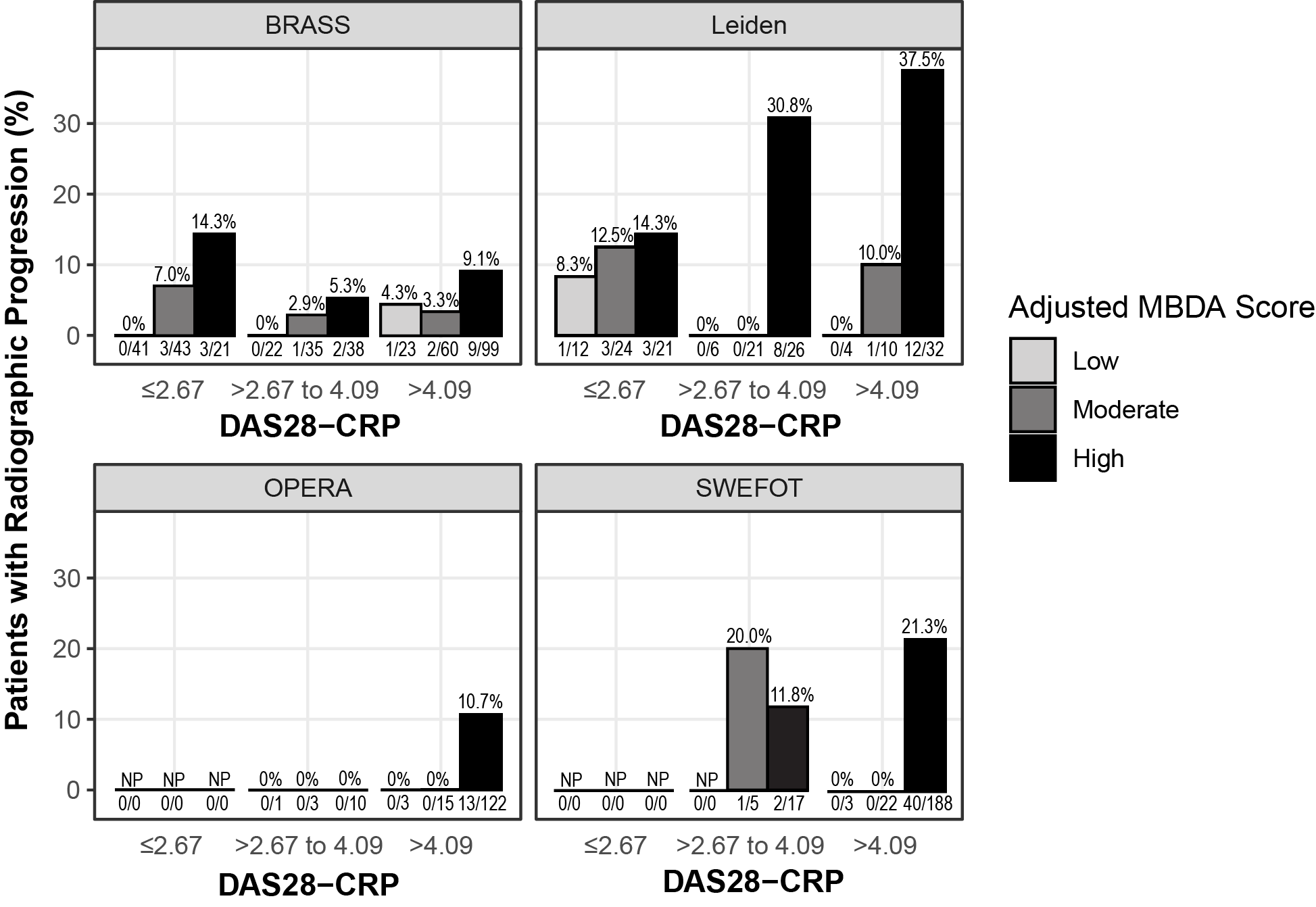

Supplement: Supplementary file 1 — Additional file 1 : Supplemental Table 1. Bivariable logistic regression predicting radiographic progression (∆TSS>5) using adjusted MBDA score with other predictors (DAS28-CRP, CDAI, SJC, seropositivity, baseline van der Heijde modified Sharp score [TSS], log[CRP+1]). Odds ratio (OR) was calculated with 95% confidence interval (CI) and p-value from likelihood ratio test. All models include a random effect on cohort. Supplemental Figure 1. Radiographic progression for patients in individual cohorts cross-classified by DAS28-CRP and adjusted MBDA score. [file 13075_2020_2389_MOESM1_ESM.docx]
